# A Warning Index Used in Prescreening for Alzheimer's Disease, Based on Self-Reported Cognitive Deficits and Vascular Risk Factors for Dementia in Elderly Patients with Type 2 Diabetes

**DOI:** 10.1155/2012/124215

**Published:** 2012-10-15

**Authors:** Toshioki Matsuzawa, Toshihiro Takata, Koichi Yokono, Hiroo Ueda, Kensuke Moriwaki, Isao Kamae, Katsuya Urakami, Takashi Sakurai

**Affiliations:** ^1^Department of General Medicine, Kobe University Graduate School of Medicine, Kobe 650-0017, Japan; ^2^Department of Metabolism, Kobe Mahoshi Hospital, Kobe 651-1242, Japan; ^3^Department of Biotatistics, Kobe University Graduate School of Medicine, Kobe 650-0017, Japan; ^4^Department of Biological Regulation, School of Health Sciences, Faculty of Medicine, Tottori University, Yonago 683-8503, Japan; ^5^Center of Comprehensive Care and Research on Memory Disorders, National Center for Geriatrics and Gerontology, Obu 474-8511, Japan

## Abstract

*Background/Aims*. Diabetes might increase the risk of Alzheimer's disease (AD). For detecting dementia, it is typical to obtain informants' perceptions of cognitive deficits, but such interviews are usually difficult in routine care. We aimed to develop a model for predicting mild to moderate AD using a self-reported questionnaire and by evaluating vascular risk factors for dementia in elderly subjects with diabetes. *Methods*. We recruited 286 diabetic and 155 nondiabetic elderly subjects. There were 25 patients with AD and 261 cognitively normal individuals versus 30 with AD and 125 normal subjects, respectively. Each participant answered subjective questions on memory deficits and daily functioning. Information on vascular risk factors was obtained from clinical charts, and multivariate logistic regression was used to develop a model for predicting AD. *Results*. The predicted probabilities used in screening for AD in diabetic subjects constituted age, education, lower diastolic blood pressure, subjective complaints of memory dysfunction noticeable by others, and impaired medication, shopping, and travel outside a familiar locality. Receiver operating characteristic analysis revealed a satisfactory discrimination for AD specific for diabetic elderly subjects, with 95.2% sensitivity and 90.6% specificity. *Conclusion*. This is the first useful index that can prescreen for AD in elderly subjects with diabetes.

## 1. Introduction

The link between vascular risk factors and dementia has recently attracted considerable attention and the impact of diabetes on a significant correlation of such factors with dementia is consistent [1–4]. However, mild cognitive dysfunction remains undetected and untreated in a considerable proportion of patients, resulting in several difficulties when treating diabetic elderly individuals. In screening for dementia-related disorders, handy cognitive tests such as the mini-mental state examination (MMSE) and the Hasegawa dementia scale-revised (HDS-R) are available. We have proposed screening indices for Alzheimer's disease (AD) using some weighted subscales of the MMSE and HDS-R, which are useful to discriminate early AD in diabetic elderly subjects [5, 6]. However, even such brief neuropsychological tests impose burdens on practitioners in ambulatory care, because the number of patients with diabetes is increasing markedly in Japan [7]. A more simple and succinct prescreening procedure is thus needed to identify individuals with high risks for cognitive decline among diabetic elderly subjects.

In the diagnostic workup for patients with dementia, it is typical to obtain information from an informant about cognitive deficits and problems in daily functioning. It has been postulated that the informant's perception of cognitive deficits correlates with objective memory testing and can differentiate between groups with and without dementia [8]. A more reliable predictor can be a discrepancy between the demented patient's and an informant's reports on cognitive status [9]. However, to obtain information on cognitive deficits from caregivers is usually difficult in the routine care of patients with diabetes. To overcome this dilemma in screening for AD in diabetic elderly subjects, we aimed to develop a disease-specific model for predicting mild to moderate AD. The first goal of this study was to administer a questionnaire about those patients' perceptions of memory problems and impaired daily functioning that are specifically evident in AD. Second, we aimed to test the association of vascular risk factors, such as hypertension, dyslipidemia, and obesity, with AD. The final goal was to propose a predictive index for AD using a comprehensive assessment of these clinical variables and to verify its propriety in nondiabetic participants.

## 2. Materials and Methods

### 2.1. Study Participants

A total of 286 diabetic patients and 155 nondiabetic individuals aged 65 or older were recruited from the outpatient's clinic of the Kobe University Hospital, Japan. The institutional review boards of Kobe University Hospital approved the research protocol, and written informed consent was obtained from each patient and his or her family members. The diabetic group had 25 subjects with mild to moderate AD and the nondiabetic group had 30. AD was diagnosed as “probable AD” from a complete medical, neuropsychological, and neuroimaging evaluation by the multidisciplinary geriatric team at each site, based on the criteria from the National Institute of Neurological and Communicative Disorders and Stroke, and the Alzheimer's Disease and Related Disorders Association [10]. In this study, mild to moderate AD was defined as a score of greater than 14 on the MMSE [11]. Patients were evaluated to rule out other causes of cognitive impairment including alcohol or drug abuse, major depression, delirium, systemic cancer, chronic infections, stroke, hypoxia, severe cardiopulmonary disorders, nutritional disorders, intracranial mass lesions, psychoses, brain trauma, or other neurological disorders including Parkinson disease or Huntington disease.

The cognitively normal participants comprised 261 and 125 participants in the diabetic and nondiabetic groups, respectively. For the primary selection of cognitive normal group, geriatric physicians, who were familiar to consultation of the demented disorders, asked the patients and their caregivers about their complaints on cognitive decline and the daily life function. Their cognitive functions were evaluated using the MMSE and a computerized test battery for AD screening [12]. They had no neurological or psychiatric disorders. After this consultation, each physician determined their cognitive status as normal.

### 2.2. Self-Reported Questionnaire for Subjective Complaints of Memory and Daily Functioning

During the first visit, one of three trained research assistants in the geriatric division assessed participants using a self-reported questionnaire that measures perception of memory deficits and impairment of activities in daily living (ADLs). The questionnaire contained three questions about each patient's perception of memory problems, which were derived from the Cambridge Examination for Mental Disorders of the Elderly (CAMDEX) interview on memory complaints [13]. The questions were as follows. (1) Do you have any complaints concerning your memory? (2) Do other people find you forgetful? (3) Do you often use notes to avoid forgetting things?

Achievement of basic and instrumental ADLs was also investigated for each individual. Basic ADLs referred to the ability to complete simple functions including walking and showering, whereas instrumental ADLs comprised more complex activities required for independent living, including grocery shopping, managing finances, meal preparation, travel outside a familiar locality, taking medication, and using public transportation [14, 15]. We assigned “1” and “0”, to “yes” and “no” answers for each question, respectively.

### 2.3. Clinical Characteristics of Diabetes and Vascular Risk Factors for Dementia

Information on diabetes and other vascular risk factors for dementia was obtained from clinical charts. Body mass index (BMI), blood pressure, levels of HbA1c, total cholesterol, triglycerides, and high density lipoprotein (HDL)-cholesterol, duration of diabetes, therapeutic use of antihyperglycemic agents and/or insulin (insulin dose and frequency of injection per day), and previous hypoglycemic episodes (number of occasions in the previous year) were investigated. The HbA1c levels are expressed in the National Glycohemoglobin Standardization Program units. The participants were also asked questions on the compliance with diet and exercise therapies (in minutes per week) by the physicians.

### 2.4. Statistical Analysis

Logistic regression analysis and *χ* ^2^ test were used to compare the demographic, subjective cognitive complaints, and vascular risk factors between subjects with AD and cognitively normal individuals in both groups. Any significant items were then entered into a multivariate logistic regression to develop a model for predicting AD, using stepwise selection with an inclusion criteria of *P* < 0.15 and exclusion criteria of *P* > 0.2 [16]. Using a developed model, a receiver operating characteristic (ROC) curve was constructed to test the relationship between sensitivity and specificity using varying cutoff points of the model for predicting AD. The area under the curve was calculated. Statistical analysis was performed using SPSS 15.0 for Windows (SPSS Inc., Chicago, IL, USA). The level of significance was set at *P* < 0.05 for all statistical analyses.

## 3. Results

### 3.1. Demographics and Clinical Profiles

The demographic and clinical features of diabetic and nondiabetic subjects are presented in Table 1. Among the subjects with diabetes, having AD was characterized by being older, being female, having a lower educational level, and having lower diastolic blood pressure compared with cognitively normal controls. Exercise was less frequently performed by patients with AD. In contrast, HbA1c levels, duration of diabetes, and lipid profiles were not significantly different between subjects with AD and cognitively normal individuals. Although pharmacological treatment of diabetes and previous hypoglycemic episodes have been reported to increase the risk of dementia [1, 17], the incidence of hypoglycemic episodes and the frequency of use of oral antihyperglycemic agents and/or insulin (insulin doses, frequency of injection) did not show any difference between the AD and cognitively normal groups. In nondiabetic participants, being female and having a lower BMI and lower diastolic blood pressure were characteristics of patients with mild to moderate AD.

The overall mean scores and ranges of the MMSE were 20.6 (14–28) and 20.8 (14–27) for AD in the diabetic and nondiabetic elderly subjects, respectively. This suggests that our subjects with AD had mild to moderate forms [11]. Cognitive status was also evaluated by a computerized neuropsychological test battery for screening AD, of which a score of 14 suggests normal cognition and one of ≤12 is associated with AD, according to the original study data of the developer [12]. The averaged scores of this cognitive test among the diabetic and nondiabetic elderly subjects were 9.0 and 9.9 for those with AD, and 14.3 and 14.4 for the cognitively normal subjects, respectively.

### 3.2. Subjective Complaints of Memory and Daily Functioning

Among three distinct questions on subjective memory complaints (Table 2), self-perception of memory dysfunction noticeable by himself/herself was not different between subjects with AD and normal controls in both diabetic and nondiabetic participants, while subjective complaint of memory deficits noticeable by others was significantly increased among the patients with AD. Responses to the question about the use of notes to avoid forgetting things tended to decrease in those with AD.

Although basic ADLs such as walking and showering were similar between the subjects with AD and the cognitively normal controls, the self-reported achievement of instrumental ADLs (grocery shopping, managing finances, meal preparation, travel outside familiar surroundings, correct use of medication, and public transportation) was significantly impaired in patients with AD among those with diabetes (Table 3). In the nondiabetic group, activities for shower, finance management, cooking, traveling, medication compliance, and use of public transport were impaired in AD.

### 3.3. Prediction of AD Using a Self-Reported Questionnaire and Risk Factors for Dementia

To develop a model for predicting AD using stepwise selection, clinical variables that were shown to be different at *P* < 0.05 (Tables 1–3) were entered into a multivariate logistic regression. This showed that advanced age (*P* < 0.001), lower education (*P* < 0.001), lower diastolic blood pressure (*P* = 0.029), memory dysfunction noticeable by others (*P* = 0.018), and impaired activity in taking medication (*P* = 0.001) were independently correlated with AD in diabetic elderly subjects. The predicted probabilities from the multivariate logistic regression analysis in screening for AD were as follows: Log *p*/(1−*p*) = 0.253*x*_1_−0.078*x*_2_−3.740*x*_3_−1.888*x*_4_ + 1.883*x*_5_−1.405*x*_6_−0.453*x*_7_−6.769; where *x*_1_ = age (years), *x*_2_ = diastolic blood pressure (mmHg), *x*_3_ = medication (yes, 1; no, 0),  *x*_4_ = shopping (yes, 1; no, 0), *x*_5_ = memory dysfunction noticeable by others (yes, 1; no, 0), *x*_6_ = travel outside familiar surroundings (yes, 1; no, 0), and *x*_7_ = education (years).

**Figure 1:**
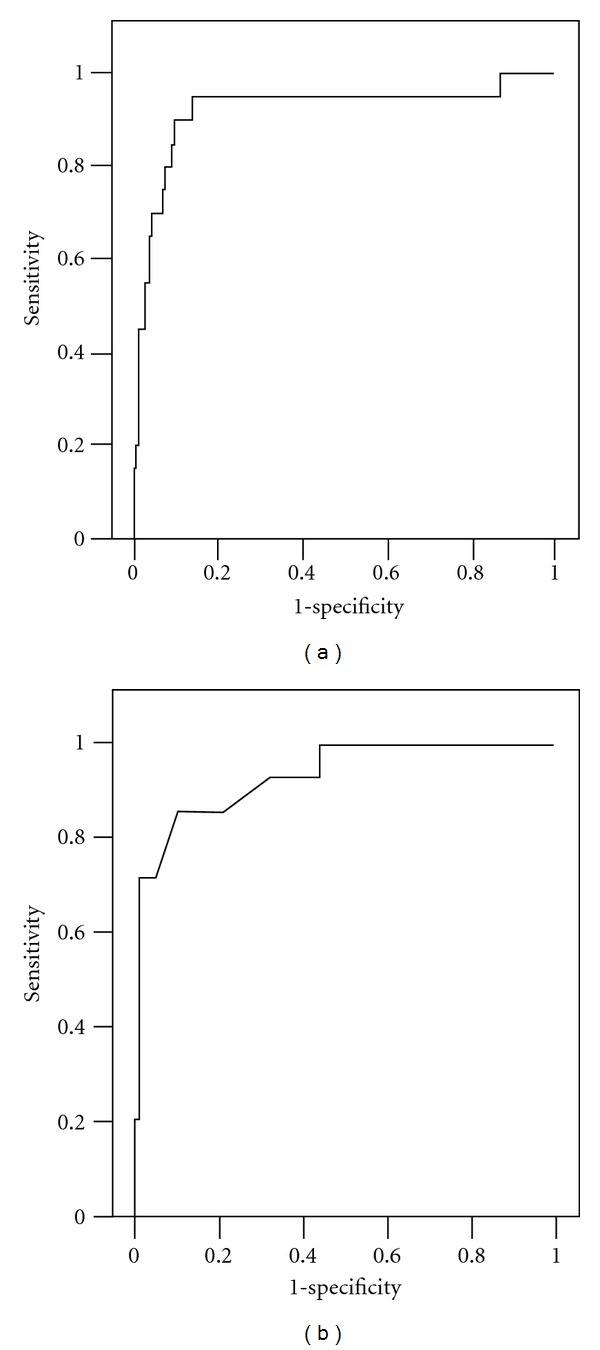
Receiver operating characteristic (ROC) curve of the best multivariate logistic regression analysis model for predicting mild to moderate Alzheimer's disease among diabetic elderly subjects (a) and nondiabetic individuals (b). The area under the curve (AUC) was 0.97 (a) and 0.93 (b).

ROC analysis revealed a satisfactory discrimination for predicting AD in diabetic elderly subjects with a sensitivity of 95.2% and a specificity of 90.6%, when the cutoff point of the model was set at 0.7888 (Figure 1(a)). When predicted probabilities from the regression analysis in diabetic participants were applied for nondiabetic subjects, a prediction of AD was discriminated with 56.7% sensitivity and 89.6% specificity.

Similarly, multivariate logistic regression revealed independent associations of lower diastolic blood pressure (*P* = 0.001) and medication compliance (*P* = 0.017) with AD in the nondiabetic group. The predicted probabilities for screening of mild to moderate AD in nondiabetic participants were found to be: Log *p*/(1−*p*) = −0.156*x*_1_−1.608*x*_2_−2.791*x*_3_−0.919*x*_4_−1.11*x*_5_+12.6; where *x*_1_ = diastolic blood pressure (mmHg), *x*_2_ = sex (male, 1; female, 0), *x*_3_ = medication (yes, 1; no, 0),  *x*_4_ = finance (yes, 1; no, 0), and *x*_5_ = travel outside familiar surroundings (yes, 1; no, 0). The ROC curve of the nondiabetic subjects was shown in Figure 1(b).

## 4. Discussion

This study demonstrates a simple but effective strategy that can be used in screening for mild to moderate AD in diabetic elderly subjects. Although the significance of an informant's perception of a patient's cognitive deficits has been emphasized previously for detecting dementia [8, 9], our results clearly indicate that comprehensive assessment of symptomatic deficits of memory and daily functioning, together with vascular risk factors for dementia, enables the discrimination of a subject developing mild to moderate AD with good sensitivity and specificity. The best prediction was obtained by the multivariate regression model that included older age, lower educational level, lower diastolic blood pressure, memory deficits noticeable by others, and impaired instrumental ADLs (shopping, medication, and travel outside familiar surroundings), which was specific for diabetic elderly subjects. For nondiabetic participants, a distinct set of variables including female gender, lower diastolic blood pressure, and impairment in dealing with finances, medication, and travel predicted AD. Our results for the first time provide a handy and succinct tool to serve as an index for predicting mild to moderate AD in elderly Japanese subjects with type 2 diabetes mellitus.

### 4.1. Subjective Complaints of Memory Deficits

This study indicates that particular questions about subjective memory complaints are predictive for AD in diabetic participants. This finding is somewhat surprising, given the rather mixed results in the literature on the value of subjective memory complaints [18]. Subjective complaints of memory deficits are often observed among subjects in the early stage of AD, but decrease gradually as the disease progresses [19]. Many studies have reported that subjective memory complaints are more often associated with depressed mood rather than cognitive impairment [8, 20, 21]. However, recent community-based studies with longitudinal designs indicate that memory complaints are predictive of cognitive decline and incident dementia, particularly in nondemented individuals with cognitive impairment, although not all studies show this association among aged persons [22–28]. Self-reported poor memory is indeed a main component of the diagnostic criteria for mild cognitive impairment [29]. In this connection, a clear definition of memory complaints might be important to explain the divergent results on their significance. Thus, this study evaluated the reliability of three different questions on subjective memory complaints originating from the CAMDEX interview [13]. Although two out of three questions on self-perception of memory deficits did not predict AD, the last question asking about memory decline noticeable by others was distinguishable even after adjustment with possible confounders, indicating the significance of particular self-reported questionnaires about subjective memory complaints when screening for AD in diabetic elderly subjects.

### 4.2. Meaning of Self-Reported Performance of Instrumental ADLs

Loss of functional, but not of basic ADLs, proved to be predictive for having mild to moderate AD. Even mild degrees of cognitive deterioration can have negative impacts on the ability to perform complex ADLs [30–32]. The completion of instrumental ADLs requires competent memory, but also involves executive functions. These entail complex cognitive abilities that enable an individual to perform tasks that include planning, problem solving, anticipation, and inhibition of irrelevant processing [33]. In a recent review by the Committee on Research of the American Neuropsychiatry Association, an expert panel suggested that measures of executive functions correlate strongly with functional capacities [34]. However, clinical assessment of functional abilities in the daily life of subjects with AD is also dependent on accurate information. Most instruments designed to assess instrumental ADLs can be influenced by the patient's personality, mood, and cognitive status [35]. Patients with AD often overestimate their functional abilities. In this respect, it should be mentioned that the capacity for self-observation is considerably preserved in patients with mild to moderate AD, although a decline in patient self-reporting on this issue is less dramatic than that seen in family reports [36, 37].

Our results indicate that among the ADL disability, impaired ability to deal with medication is the most predictive for AD in both diabetic and nondiabetic individuals. Besides impaired activity for travel outside familiar surroundings, shopping activity was specifically involved with AD in the diabetic participants, and financial ability in the nondiabetic subjects. Shopping and managing finances are classified in the identical subdomain of functional ADLs that correspond to the identical staging of dementia [38]. Errors in shopping tasks are more likely to be associated with decrements in visual searching skills, selective attention, and rapid information processing [39]. Impaired attention and decreased information processing speed have been reported in aged persons with type 2 diabetes [40].

### 4.3. Vascular Risk Factors for Predicting AD

There is a growing consensus that vascular disease may exacerbate or contribute to the manifestation of symptoms in subjects with dementia [41]. Barnes et al. [42] have reported that a late-life dementia risk index, composed of age, cognitive test performance, body mass index, apolipoprotein E *ε*4 alleles, cerebral white matter disease, ventricular enlargement, internal carotid artery thickening, history of bypass surgery, slow physical performance, and lack of alcohol consumption, can accurately stratify older adults into those with low, moderate, and high risk of developing dementia. It has been postulated that midlife high blood pressure is a risk for late-life cognitive impairment and dementia, and low diastolic pressure in older adults might be associated with the subsequent development of dementia and AD [43, 44]. Accelerated atherosclerosis and low perfusion of cerebral blood flow in diabetic elderly subjects could be implicated in a mechanism of how abnormal blood pressure affects the onset of dementia. In contrast, no predictive power for other aspects of diabetes, including HbA1c level, lipid abnormalities, obesity, hypoglycemia, or treatment modality was found in the present study [1, 17].

### 4.4. Limitation and Strength

This study had several limitations. There were several biases in the selection of our participants, who were treated in the outpatient clinic of the Kobe University hospital. They tended to have serious diabetic complications and other morbidities, while they were also motivated for treatment of their illness. Despite this, our data might represent a best-case scenario for practitioners because we approached many physicians treating diabetes in aged persons. In addition, the effects of depressive mood on cognitive status were not evaluated, although patients with major depression were excluded from our participants. Finally, it might be possible that cognitive normal subjects include some individuals with mild cognitive impairment, because detailed cognitive tests were not performed in the cognitive normal group. However, geriatric physicians, who were familiar to consultation of the demented disorders, asked the patients and their caregivers about their complaints on cognitive decline and the daily life function. Their cognitive functions were evaluated using the MMSE and a computerized test battery for AD screening. After this consultation, each physician determined their cognitive status as normal.

On the other hand, the present study had several strengths. The advantage of screening for mild to moderate AD using this discriminating index has been clearly demonstrated in diabetic elderly subjects. Second, the model for predicting AD is so succinct and easily available that nonclinical staff in outpatient clinics could administer it with ease. This would greatly improve the burden of practitioners who must face several clinical problems in elderly patients with diabetes. When considering a total scheme for detecting AD in diabetic elderly subjects, high-risk individuals can be selected using this warning index for AD. Such persons can then continue to a secondary evaluation using brief cognitive tests such as the MMSE and HDS-R [5, 6] and ultimately consult with specialists for dementia-related disorders.

## 5. Conclusions

We have proposed the importance of this maneuver in prescreening for AD, using this self-reported questionnaire and including vascular risk factors in the model. This approach is also applicable for nondiabetic elderly subjects. The clinical relevance of this index aimed at prescreening for AD should be validated by further investigations.

## Figures and Tables

**Table 1:** Demographic and clinical profile of study participants.

	Diabetic subjects		Nondiabetic subjects	
	AD	CN	AD	CN
Number	25	261	30	125
Age (years)	78.6 (5.8)*	72.7 (5.6)	77.5 (6.3)	72.6 (4.6)
Female (%)	68.0*	50.2	80.0^#^	50.4
Education (years)	9.3 (2.0)*	11.9 (3.0)	11.0 (2.5)	12.5 (3.0)
BMI (kg/m^2^)	23.2 (4.6)	23.0 (3.4)	21.7 (2.2)^#^	23.6 (4.2)
Systolic blood pressure (mmHg)	127.0 (16.0)	130.0 (13.3)	126.6 (17.3)	128.6 (15.7)
Diastolic blood pressure (mmHg)	60.0 (15.1)*	70.0 (9.4)	58.5 (16.0)^#^	72.0 (9.0)
Total cholesterol (mg/dL)	197.8 (43.7)	206.1 (30.0)	214.4 (34.1)	196.1 (29.8)
Triglyceride (mg/dL)	118.9 (49.3)	157.4 (83.7)	131.0 (61.6)	109.8 (54.0)
HDL-cholesterol (mg/dL)	60.2 (12.5)	53.4 (14.7)	69.5 (18.6)	60.9 (17.9)
HbA1c (%)	7.1 (0.8)	7.2 (1.1)	—	—
Duration of diabetes (years)	16.6 (9.9)	14.6 (10.7)	—	—
Hypoglycemic episodes (times/year)	4.5 (13.0)	11.4 (47.2)	—	—
Insulin use (%)	40.0	30.3	—	—
Insulin dose (U/day)	17.0 (11.6)	22.7 (12.9)	—	—
Insulin injection (times/day)	2.4 (1.0)	2.4 (1.0)	—	—
Oral hypoglycemic agent use (yes = 1, no = 0)	0.8 (0.4)	0.7 (0.5)	—	—
Exercise (minutes/week)	72.3 (12.9)*	221.9 (468.7)	209.3 (161)	225.8 (208.7)
Diet therapy compliance (very poor = 1, poor = 2, normal = 3, good = 4)	3.2 (0.9)	3.3 (0.6)	3.3 (0.7)	3.4 (0.8)
History of heart disease (%)	4.0	19.9	3.7	20.0
History of cerebrovascular disease (%)	12.0	10.0	6.7	8.0
MMSE (score)	20.6 (3.8)*	28.0 (0.0)	20.8 (3.7)^#^	27.4 (2.1)
Computer-based screening test (score)	9.0 (2.9)*	14.3 (0.5)	9.9 (2.9)^#^	14.4 (0.5)

Values are the mean and (SD) and percentages. *P < 0.05 and ^#^ *P* < 0.05 compared with cognitively normal subjects in the diabetic and nondiabetic groups, respectively. AD: Alzheimer's disease; CN: cognitively normal; BMI: body mass index; MMSE: mini-mental state examination.

**Table 2:** Subjective complaints of memory deficits.

	Diabetic subjects		Nondiabetic subjects	
	AD	CN	AD	CN
Do you have any complaints concerning your memory? (yes, %)	64.0	70.1	83.3	67.2
Do other people find you forgetful? (yes, %)	60.0*	29.9	60.0^#^	30.4
Do you often use notes to avoid forgetting things? (yes, %)	72.0*	82.3	73.3	86.4

Values are the percentages of “yes” answer to each question. Comparison of subjective cognitive complaints between subjects with AD and cognitively normal subjects was performed by using the *χ* ^2^ test. *P < 0.05 and ^#^ *P* < 0.05 compared with cognitively normal subjects in the diabetic and nondiabetic groups, respectively. AD: Alzheimer's disease; CN: cognitively normal.

**Table 3:** Self-reporting of basic and instrumental activities of daily living (ADLs).

	Diabetic subjects		Nondiabetic subjects	
	AD	CN	AD	CN
Basic ADL				
Walking (able, %)	80.0	90.0	80.0	89.6
Shower (able, %)	92.0	97.8	80.0^#^	96.8
Instrumental ADL				
Grocery shopping (able, %)	80.0*	96.1	90.0	97.6
Managing finances (able, %)	68.0*	96.2	73.3^#^	96.8
Meal preparation (able, %)	76.0*	91.2	90.0	96.0
Travel outside familiar surroundings (able, %)	48.0*	95.0	50.0^#^	95.2
Medication compliance (able, %)	48.0*	96.2	63.3^#^	95.2
Ability to use public transport (able, %)	76.0*	96.2	83.3^# ^	96.0

Values are the percentages of “able” answer to each life function. Comparison of ADLs between subjects with AD and cognitively normal subjects was performed by using the *χ* ^2^ test. *P < 0.05 and ^#^ *P* < 0.05 compared with cognitively normal subjects in the diabetic and nondiabetic groups, respectively. AD: Alzheimer's disease; CN: cognitively normal.
